# Model-Based Prediction to Evaluate Residence Time of Hyaluronic Acid Based Dermal Fillers

**DOI:** 10.3390/pharmaceutics13020133

**Published:** 2021-01-21

**Authors:** Hyo-jeong Ryu, Seong-sung Kwak, Chang-hoon Rhee, Gi-hyeok Yang, Hwi-yeol Yun, Won-ho Kang

**Affiliations:** 1 Gwangkyo R & D Center, Medytox Inc., Suwon 16506, Korea; hjryu5767@gmail.com (H.-j.R.); sskwak@medytox.com (S.-s.K.); yanggh@medytox.com (G.-h.Y.); 2 College of Pharmacy, Chungnam National University, Daejeon 34134, Korea; 3 Osong R & D Center, Medytox Inc., Cheongju 28126, Korea; chrhee@medytox.com

**Keywords:** HA-based dermal filler, kinetic model, swelling, degradation, prediction, NONMEM

## Abstract

Dermal fillers are gel-type substances for nonsurgical medical-device use to achieve facial rejuvenation. Currently, the most widely used skin fillers are hyaluronic-acid-based dermal fillers. This study aimed to explain the change in the volume of injected dermal fillers by developing a mathematical kinetic model for various dermal fillers. The kinetics of the injected fillers were separated by a biphasic phenomenon. We attributed an increase in filler volume to the hydration of hyaluronic acid molecules and injection-site reaction and a decrease in volume to enzyme-mediated degradation. To explain these in vivo characteristics of dermal fillers, we proposed a two-compartment model, divided into a depot compartment (where the filler was injected) and a subcutaneous compartment (an observation compartment where the fillers swell and degrade), assuming that the swelling and degradation occurred in accordance with the swelling and degradation rate constants, respectively. The model was developed using five hyaluronic-acid-based dermal fillers and NONMEM. We determined that the rate-limiting step for the complete degradation of the dermal fillers in vivo was the swelling phase, as described by the swelling rate constant (*K_swell_*). This study could enable scientists developing novel dermal fillers to predict the in vivo behavior of fillers.

## 1. Introduction

Over the last 20 years, interest in dermal fillers as a nonsurgical medical device for facial rejuvenation has significantly increased. The growing public awareness and demand for dermal fillers as injectable implants drive the market growth. Approximately 160 dermal filler products, produced by more than 50 different manufacturers, are currently available worldwide [1,2].

A dermal filler is a gel-type substance that is injected under the skin. It is composed of various natural or synthetic substances. Dermal fillers have been classified according to the product composition; the primary ingredients include collagen, synthetic hyaluronic acid (HA), poly-l-lactic acid, calcium hydroxyapatite, polymethyl methacrylate, and polyacrylamide gel. Currently, the most widely used skin fillers are HA-based dermal fillers because of their excellent safety, ease of application, and satisfactory aesthetic results.

HA is a naturally occurring high molecular weight polysaccharide, composed of repeating disaccharide units of *N*-acetyl-glucosamine and *D*-glucuronic acid, which form an essential component of the skin and connective tissues. HA has been widely used for facial rejuvenation, as it is easy to use, non-immunogenic, non-carcinogenic, and biocompatible [3]. It’s in vivo degradation occurs primarily through enzymatic degradation and free radical oxidation [4]. In 2015, HA-based dermal fillers accounted for more than 92% of all filler treatments in the US market. These fillers consist of long chains of HA that are usually chemically modified by cross-linking [1,2,5,6,7,8,9]. Cross-linking density affects the degradation lifetime of HA hydrogel. Hence, the cross-linking technology used determines the performance of the dermal filler. Besides 1,4-butanediol diglycidyl ether (BDDE), which has been mainly used for HA’s covalent binding, other chemical cross-linkers have been used, such as glutaraldehyde, hexamethylenediamine, poly(ethylene glycol) digycidyl ether, divinyl sulfone, carbodiimide, adipic acid dihydrazide, and 2,7,8-diepoxyoctane. More recently, HA hydrogels have been cross-linked using novel bis(β-isocyanatoethyl) disulphide (BIED). This HA hydrogel has good biocompatibility and no cytotoxicity; moreover, it can act as an inflammatory and immune modulator. Therefore, HA-based hydrogels have potential medical applications depending on the type and process of chemical cross-linking [10,11]. In humans, HA-based dermal fillers for volumizing and contouring are known to retain their effects for a few months to a year or more. In a rodent model, the effects of a subcutaneously administered dermal filler (Juvederm^®^ Vista ULTRA PLUS, Allergan Plc., Dublin, Ireland) were maintained for 64 weeks and up to 18 months [12].

Nonclinical investigation of dermal filler products includes filler performance tests to evaluate their effectiveness (such as contouring, volumizing, and volume maintenance effects) before clinical trials. Generally, these studies evaluate the residual volume after subcutaneous or intradermal injection in hairless mice. During the initial phase of treatment after filler injection, the volume at the injection site increases above the injected volume; this phenomenon is called swelling. It is followed by a steady decrease in volume over the mid–late phase of the treatment. Swelling occurs due to injection site reaction on the skin and hydration of HA molecules. The volume decrease during the mid–late phase of the treatment results from degradation mediated by endogenous hyaluronidase [13,14,15,16].

The International Organization for Standardization (ISO) 10993-6 (Biological evaluation of medical devices—Part 6: Tests for local effects after implantation) recommends the utilization of a minimum of 3 observation time intervals for medical devices such as dermal fillers: (1) early time frame when there is no or minimal degradation; (2) middle time frame when degradation is occurring; and (3) late time frame, when minimal amounts of the absorbable components remain at the implant site, to evaluate the degradation of absorbable materials [17,18]. This guideline additionally recommends considering relevant information from in vitro degradation studies and conducting a pilot study in rodents for establishing an adequate test period in the main study. However, the complete degradation of the dermal filler in a rodent model is difficult to evaluate because the lifespan of rodents is limited and this study requires a considerable amount of observation time and effort. Thus, a mathematical model was needed to predict the time for complete degradation of fillers, based on rodent model data.

Although the ISO guideline states that in vitro degradation studies or mathematical modeling can be helpful to estimate the degradation time point [17,18], it does not provide details regarding these methods. Furthermore, no previous studies have reported mathematical modeling approaches and in vitro to in vivo extrapolation methods for this purpose. Therefore, several researchers evaluating the biological characteristics of dermal fillers are currently in need of such mathematical approaches.

The objective of this study was to develop a mathematical kinetic model for five HA dermal fillers from different companies (99 fill^®^, Hanmi Pharmaceutical Co., Ltd., Seoul, Korea; Juvederm^®^ VOLUMA with Lidocaine, Allergan Plc.; Neuramis^®^ VOLUME Lidocaine, Medytox Inc., Cheongju-si, Korea; Restylane^®^ Lyft with Lidocaine, Galderma S.A., Lausanne, Switzerland; and YVOIRE^®^ contour plus, LG Chem Ltd., Seoul, Korea) and to apply this model to simulate the volume–time profile of the fillers to predict the time for complete degradation. The properties of HA dermal fillers are provided in Supplementary Materials Table S1. The in vivo characteristics and complete degradation time of five different dermal fillers were successfully predicted using the mathematical kinetic model developed in this study.

## 2. Materials and Methods

### 2.1. Animals and Study Design

All experimental procedures were approved by the Institutional Animal Care and Use Committee of Medytox Inc. (Approval No. A-2018-010, Approval Date. 20 July 2018) before study initiation. Six- to seven-week-old female hairless mice (SKH1-Hr^hr^) were purchased from Orient Bio, Inc. (Seongnam-si, Korea) and housed in individually ventilated cage (IVC) racks with free access to standard rodent feed (R+40RMM-10, SAFE, Augy, France) and water. The animals were placed under a controlled environment with a 12 h light/dark cycle at 23 ± 3 °C and relative humidity of 55 ± 15%. The dermal filler was administered subcutaneously at a dose of 100 μL in the dorsal skin of the hairless female mice (*n* = 8 mice per group). After anesthetic injection in each mouse, the filler volume was measured using a PRIMOS Lite system (Canfield Scientific Inc., Parsippany-Troy Hills, NJ, USA) at 0, 1, 4, 7, 21, and 28 days and monthly from 2 to 18 months. The filler image was recorded using a PRIMOS 5.8E system (Canfield Scientific Inc. Parsippany-Troy Hills, NJ, USA) on day 0 and at the other time points. The change in volume and height was quantitatively determined according to the 3D (3 dimensional) analysis for the filler image under the skin. The minimum detectable value for the filler volume was 3 mm^3^ (0.003 cm^3^). The PRIMOS system is a fast and accurate method for measuring filler volume in humans and animals [19,20,21,22].

### 2.2. Establishment of Kinetic Model for Dermal Fillers

**Figure 1:**
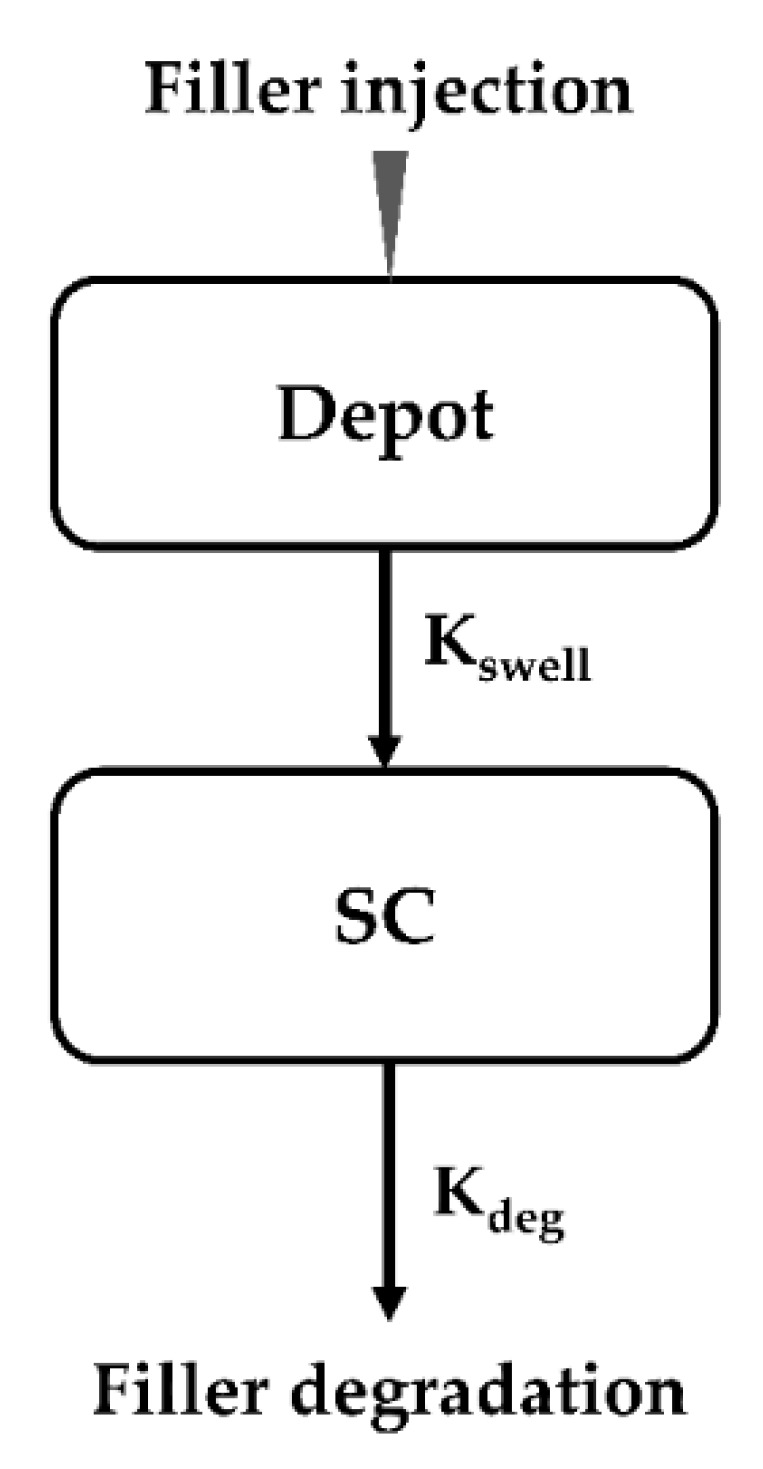
The schematic kinetic model for dermal fillers; DEPOT, Depot compartment; SC, Subcutaneous compartment, *K_swell_*, Swelling rate constant; *K_deg_*, Degradation rate constant.

Kinetic modeling for the dermal fillers was performed using NONMEM version 7.4 (ICON Development Solutions, Ellicott City, MD, USA) with the assistance of Pirana (ver. 2.9.8, Princeton, NJ, USA) and PsN (ver. 4.9.0, Husargatan, Uppsala, Sweden). Statistical and graphical analyses were performed using R (ver. 3.6.1, Welthandelsplatz, Vienna, Austria), R Studio (ver. 1.2.1335), MS Excel 2016, or GraphPad Prism (ver. 7.05). Following the visual assessment of the volume–time profiles of the five fillers, the one-compartment degradation model with first-order swelling were evaluated as the kinetic model to conduct the analysis and prediction of the time for complete decomposition (T_cd_) of the structural fillers. The kinetic model structure is described in Figure 1.

In the kinetic model for these fillers, the depot compartment (DEPOT) represented the injection site of the filler, and the subcutaneous compartment (SC) represented the subcutaneous volume. Although the injected site was also a subcutaneous site, the compartments were separated to analyze the initial SC volume and the change in volume through the two processes of swelling and biodegradation. Hence, the kinetic model of the fillers consisted of two rate constants, which were parameterized in terms of the swelling rate constant (*K_swell_*) and degradation rate constant (*K_deg_*) using ADVAN6 and the first-order conditional estimation method with interaction (FOCE-I). Since enzyme reactions in the body follow first-order kinetics, *K_swell_* and *K_deg_* were assumed as first-order rate constants. The inter-individual variability (IIV) of each rate constant was evaluated using an exponential method. The IIV of *K_deg_* was explained by the multiplication of the IIV of *K_swell_* and the slope obtained with the IIVs of each rate constant because there was a strong linear relationship between the IIVs of each rate constant. Residual variability (RV) was evaluated using a proportional error model. Each rate constant of the dermal filler kinetic model was assumed as log-normal distribution and described as Equations (1) and (2).

(1) $$K_{swell\;}\left(day^{-1}\right)=\;theta_{1}\;\cdot \;exp^{eta_{1}}$$

(2) $$K_{deg\;}\left(day^{-1}\right)=\;theta_{2}\;\cdot \;exp^{theta_{3}\;\cdot \;eta_{1}}$$

The differential equations used to describe the final dermal filler kinetic model were as follows (Equations (3) and (4)).

(3) $$\frac{dDEPOT}{dt}=\;-K_{swell}\;\times DEPOT$$

(4) $$\frac{dSC}{dt}=\;K_{swell}\;\times DEPOT-\;K_{deg}\;\times SC$$

where *K_swell_* represents the first-order swelling rate constant for the filler volume from the DEPOT to the SC compartment and *K_deg_* represents the first-order degradation rate constant for the filler volume. The units of the filler volume injected into the DEPOT compartment and the observed residual volume in the SC compartment coincided in cm^3^.

### 2.3. Model Diagnostics and Evaluation

A visual predictive check (VPC) was conducted for the evaluation of the final kinetic model for the fillers. Using the final model, 1000 simulated replicates of the original dataset were generated, and the 5th percentile, median, and 95th percentile calculated from the simulated residual volume were compared to the observed residual volume of the remaining filler. In addition, bootstrap analysis was performed as an internal model evaluation. The final model parameters were compared to 95% confidence intervals, which were calculated using the 2.5 and 97.5 percentiles obtained from 1000 bootstrapping.

### 2.4. Simulation for Expected Time for Complete Decomposition

Monte–Carlo simulations (*n* = 1000) were performed using the estimates for *K_swell_*, *K_deg_*, and IIV on *K_swell_* and the (RV) of the final model for the fillers to predict the T_cd_ of various dermal fillers. Following the NONMEM simulation, we calculated 90% prediction intervals from the simulation data and subsequently plotted the median and 90% prediction interval (5% to 95% quantile) using dplyr and ggplot2 of the R package. Because the lowest detection limit of the PRIMOS 5.8E system used in this animal study was 3 mm^3^ (0.003 cm^3^), we could determine T_cd_ as the time in which the simulated median volume reached below 3 mm^3^ (0.003 cm^3^).

## 3. Results

### 3.1. Volume Change of Fillers after Injection in Hairless Mice

**Figure 2:**
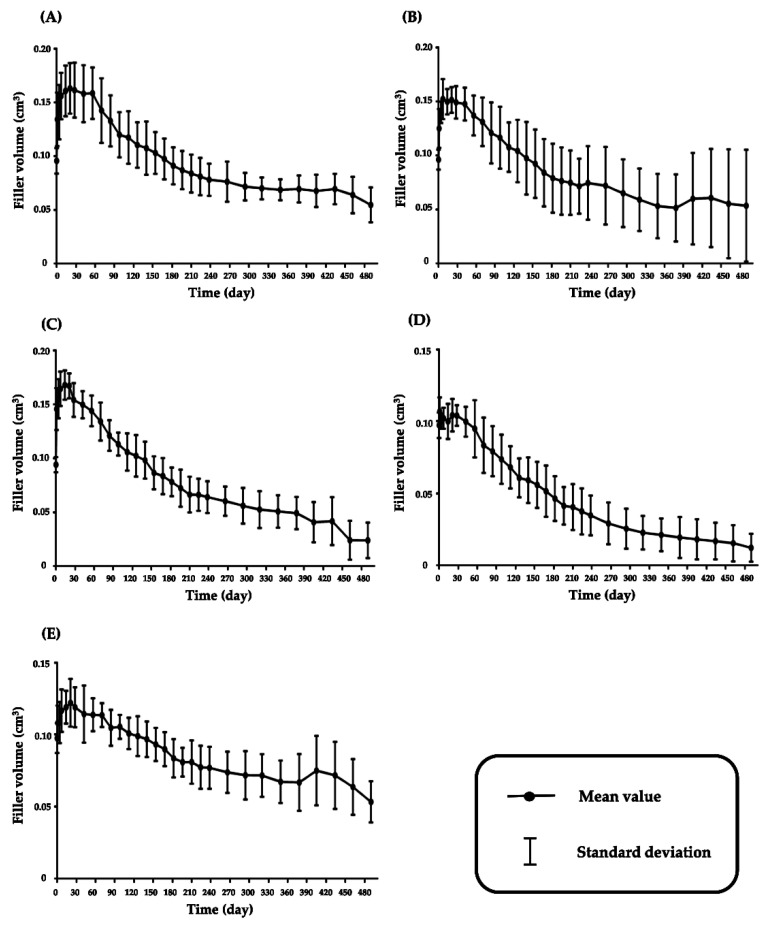
Mean volume-time profiles of 5 hyaluronic acid (HA) dermal fillers after injection in hairless mice, (**A**) 99 fill^®^; (**B**) Juvederm^®^ VOLUMA with Lidocaine; (**C**) Neuramis^®^ VOLUME Lidocaine; (**D**) Restylane^®^ Lyft with Lidocaine; (**E**) YVOIRE^®^ Contour plus.

The changes in the volume of the fillers with respect to time were determined, in compliance with ISO 10993-6 [18]. The volumes in the hairless mice after injection of the fillers at 100 μL are shown in Figure 2. The volume of the fillers increased for up to 2 months and gradually decreased over time from 2 months to 18 months.

### 3.2. Kinetic Modeling for Swelling and Degradation

**Figure 3:**
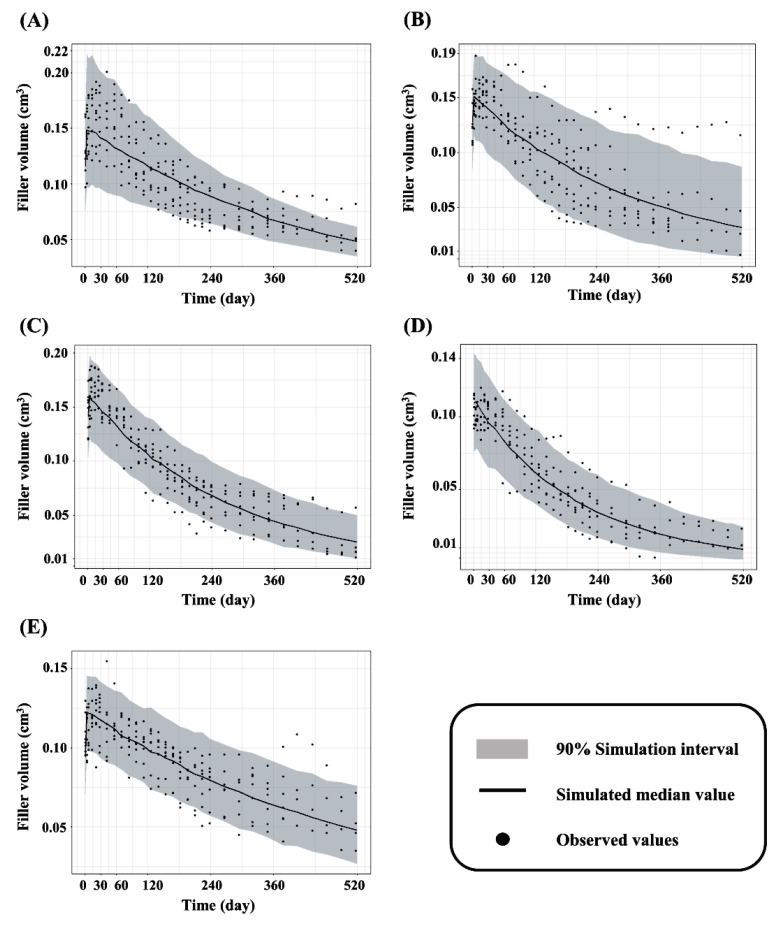
Visual predictive check for the final filler’s models of (**A**) 99 fill^®^; (**B**) Juvederm^®^ VOLUMA with Lidocaine; (**C**) Neuramis^®^ VOLUME Lidocaine; (**D**) Restylane^®^ Lyft with Lidocaine; and (**E**) YVOIRE^®^ Contour plus.

The dataset for the five marketed dermal fillers were included in the analysis. The observed residual volume from the volume–time profiles were best described by a one-compartment degradation model with first-order swelling. The estimated *K_swell_* and *K_deg_* values from the final kinetic model for each of the five dermal fillers are summarized in Table 1. The values were similar to those generated from the bootstrap replications, indicating good precision in the final models. Figure S1 shows basic goodness-of-fit plots for the final model of each dermal filler. The individual and population predictions were evenly distributed across the line of identity, indicating a good model fit. VPCs with 95% prediction intervals using the final kinetic model are shown in Figure 3. The VPC plots indicate that most of the observed residual filler volume values were within 95% of the prediction interval of the simulated data. The results indicate that the predictive performance is adequate for the final kinetic model of the fillers.

### 3.3. Simulation for Expected Time for Complete Decomposition of Dermal Fillers

**Figure 4:**
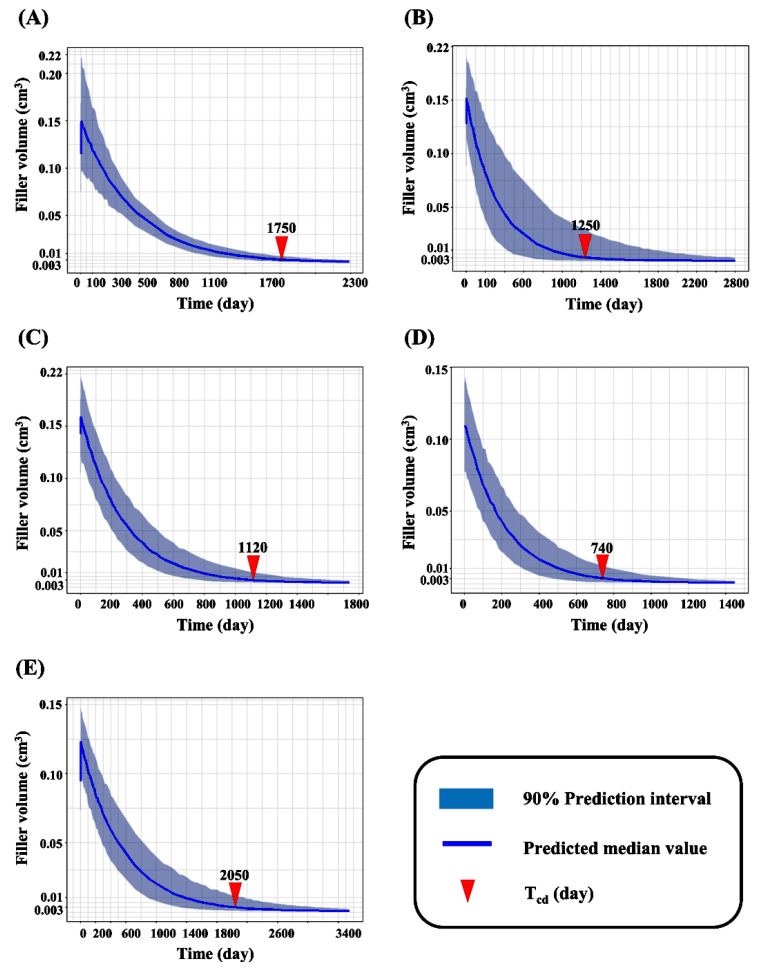
The Simulated complete decomposition time of (**A**) 99 fill^®^; (**B**) Juvederm^®^ VOLUMA with Lidocaine; (**C**) Neuramis^®^ VOLUME Lidocaine; (**D**) Restylane^®^ Lyft with Lidocaine; (**E**) YVOIRE^®^ Contour plus. T_cd_, complete decomposition time.

The time for complete decomposition (T_cd_) of the fillers was predicted by simulation; the data are summarized in Table 2. The T_cd_ values for 99 fill^®^, Juvederm^®^ VOLUMA with Lidocaine, Neuramis^®^ VOLUME LIDOCAINE, Restylane^®^ LYFT with Lidocaine, and YVOIRE^®^ contour plus were estimated to be 1750 days with 45% variation as an IIV or RV, 1250 days with over 100% variation, 1120 days with approximately 60% variation as IIV and RV, 740 days with 90% variation as IIV and RV, and 2050 days with approximately 80% variation as IIV and RV, respectively (Figure 4).

## 4. Discussion

Mathematical kinetic models were developed for five HA dermal fillers and evaluated with respect to the rate constants of swelling and degradation of each dermal filler. Generally, the decomposition of cross-linked hydrogels, such as HA-based dermal filler is caused by two mechanisms: hyaluronidase degradation and free radical degradation [23,24]. Hyaluronidase and free radical-mediated degradation is the natural elimination process of HA filler because of constant exposure to endogenous hyaluronidase and free radical oxidation [4,25]. The swelling of HA dermal filler is known to follow first-order kinetics in in vitro swelling tests, in accordance with the following equations [26,27,28,29,30].

(5) $$Q=\;\frac{W_{t}-\;W_{0}}{W_{0}}$$

(6) $$\frac{dQ_{t}}{dt}=k\;\cdot \left(Q_{e}-\;Q_{t}\right)$$

where *Q*, *W*_0_, *W_t_*,, *Q_e_*, *Q_t_*, and *k* indicate the swelling ratio, dry (dehydrated) weight of the hydrogel, wet (hydrated) weight of the hydrogel at time *t*, swelling ratio when the hydrogel reaches the maximum volume or equilibrium, swelling ratio at time t, and proportionality constant between the rate of swelling and the unrealized swelling capacity, respectively. Hence, after injection of the dermal fillers, the kinetics model was well explained using a one-compartment degradation model with first-order swelling. In addition, a population approach including IIV and RV was conducted to minimize variability originated from skin injection on subjects; subsequently, the kinetics of the dermal fillers was well implemented based on the evaluation of visual and numerical criteria such as a decrease in objective function value (OFV), goodness-of-fit plots, VPC, and bootstrapping.

**Figure 5:**
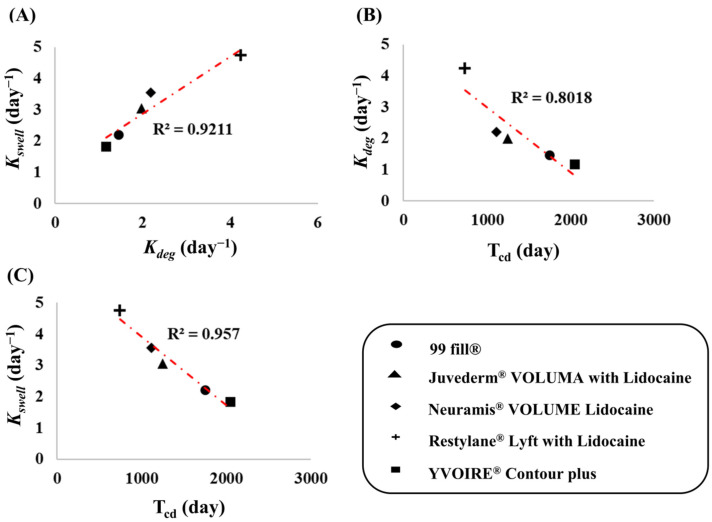
The relationship among the estimated and simulated parameters (*K_swell_*_,_ *K_deg_*_,_ T_cd_) from the 5 HA dermal filler’s kinetic model, (**A**) *K_deg_* vs. *K_swell_;* (**B**) T_cd_ vs *K_deg_*; (**C**) T_cd_ vs. *K_swell_*_._ *K_swell_*, swelling rate constant; *K_deg_*_,_ degradation rate constant; T_cd,_ complete decomposition time.

Additionally, the relationships among the three parameters (*K_swell_*, *K_deg_*, and T_cd_), as presented in Figure 5, were estimated (*K_swell_* and *K_deg_*) and simulated (T_cd_). According to these relationships among the five filler products, *K_swell_* exhibited a strong linear relationship with *K_deg_*, indicating that fillers with faster swelling behavior degrade faster and vice versa. Because both parameters were strongly related to T_cd_, fillers that swell and degrade faster were also associated with a shorter T_cd_. Therefore, the rate-limiting step for T_cd_ was suggested to be the swelling phase, as described by *K_swell_*, based on the abovementioned relationships and the order of the physiological reaction in the body (*K_swell_*, *K_deg_*, and T_cd_).

Generally, dermal fillers can be categorized as bi-phasic and mono-phasic HA fillers based on the differences in their formulations. The material properties of each dermal filler are listed in Table S1. Bi-phasic fillers contain a mixture of cross-linked HA and non-cross-linked HA used as a carrier. Mono-phasic HA fillers are produced through various degrees of cross-linking by varying the amount of high and low molecular weight HA [31,32,33]. In this study, two types of dermal fillers (monophasic fillers (99 fill^®^, Juvederm^®^ VOLUMA with Lidocaine, and Neuramis^®^ VOLUME Lidocaine) and bi-phasic fillers (Restylane^®^ Lyft with Lidocaine and YVOIRE^®^ contour plus)) were applied with the same structural mathematical model. There was a considerable difference from the modeling results between mono-phasic and bi-phasic fillers. The ratio of *K_swell_*_,_ to *K_deg_* was approximately 1.5–1.6 for the mono-phasic fillers; however, the ratio was approximately 1.1 for the bi-phasic fillers. This difference in the ratio indicated that the swelling rate of the mono-phasic fillers was considerably faster than that of the bi-phasic fillers and that the characteristics of the mono- and bi-phasic HA fillers were reflected in the parameter estimation step. Therefore, the final structural model sufficiently explained the kinetics of each HA filler based on the characteristics of the HA types. In addition, other animal studies have reported that the degree of swelling was more pronounced in monophasic fillers than in biphasic fillers [16,34]. We also explored the other correlations between the estimated parameters and each property of HA filler (e.g., molecular weight); however, there were no meaningful relationships, except gel type.

The simulation results of our study revealed that the T_cd_ of most fillers exceeded the lifespan or was close to the maximum lifespan of animals used in experiments. However, it is important to predict T_cd_ in the early stages of any animal experiment to assess dermal filler performance and determine the adequate time for histopathological evaluation in GLP studies, in accordance with the ISO guidelines.

This study had some potential limitations. First, the results of this study were obtained only for HA-based dermal fillers. The mathematical kinetic model developed by us does not reflect the kinetics of semi-permanent or permanent fillers; therefore, our model should be utilized for HA-based dermal fillers. Second, in this study, the in vivo prediction of filler characteristics was conducted with only rodent data. Further studies in mini-pigs, the skin structure of which is similar to human skin structure, would be beneficial in predicting dermal filler characteristics in humans.

In conclusion, we present a new approach to predict the in vivo behavior of dermal fillers using rodent data and pharmacometrics. This approach would be beneficial for analyzing the early, middle, and late phases of dermal filler degradation as recommended by ISO 10993-6.

## Figures and Tables

**Table 1:** Parameter estimates of the final kinetic model after subcutaneous injection of 5 HA dermal fillers to hairless mouse and results of bootstrap validation (*n* = 1000).

Trade Name	Parameters	Estimates (%RSE)	IIV (%RSE)	Bootstrap Median (2.5–97.5% Percentile)
99 fill^®^	*K_swell_* (day^−1^)	2.2 (12. 6%)	17.9% (20.3%)	2.24 (1.84–2.85)
	*K_deg_* (day^−1^)	1.45 (8.8%)	-	1.46 (1.26–1.73)
	Slope	0 *	-	-
	Proportional residual variability CV%	16.3% (13.1%)	-	-
Juvederm^®^ VOLUMA with Lidocaine	*K_swell_* (day^−1^)	3.04 (17.1%)	46.5% (30.9%)	3.06 (2.10–4.03)
	*K_deg_* (day^−1^)	1.98 (19.6%)	-	1.99 (1.30–2.74)
	Slope	1.15 (2.8%)	-	1.15 (1.07–1.41)
	Proportional residual variability CV%	13.8% (12.3%)	-	-
Neuramis^®^ VOLUME Lidocaine	*K_swell_* (day^−1^)	3.55 (8.5%)	22.3% (13.5%)	3.54 (3.03–4.19)
	*K_deg_* (day^−1^)	2.2 (8.8%)	-	2.19 (1.87–2.63)
	Slope	1.06 (11.1%)	-	1.05 (0.754–1.33)
	Proportional residual variability CV%	14.9% (14%)	-	-
Restylane^®^ Lyft with Lidocaine	*K_swell_* (day^−1^)	4.74 (13.1%)	29% (22.3%)	4.79 (3.87–5.90)
	*K_deg_* (day^−1^)	4.24 (11.6%)	-	4.25 (3.52–5.23)
	Slope	1.01 (18.3%)	-	1.02 (0.436–1.53)
	Proportional residual variability CV%	17.9% (11.9%)	-	-
YVOIRE^®^ Contour plus	*K_swell_* (day^−1^)	1.82 (10.2%)	25% (38.5%)	1.84 (1.50–2.25)
	*K_deg_* (day^−1^)	1.47 (10.7%)	-	1.49 (1.20–1.83)
	Slope	1.16 (14.5%)	-	1.19 (0.891–2.88)
	Proportional residual variability CV%	11.2% (10.8%)	-	-

* The slope for 99 fill^®^ was fixed as 0 because of the estimation tendency to zero value.

**Table 2:** Parameter estimates of the final kinetic model and results of the Monte Carlo simulation (*n* = 1000).

Trade Name	T_cd_ Median (5–95% Quantile)
99 fill	1750 day (1450~2220 day)
Juvederm^®^ VOLUMA with Lidocaine	1250 day (630~2800 day)
Neuramis^®^ VOLUME Lidocaine	1120 day (760~1630 day)
Restylane^®^ Lyft with Lidocaine	740 day (490~1190 day)
YVOIRE^®^ Contour plus	2050 day (1360~3130 day)

Kswell, swelling rate constant; Kdeg, degradation rate constant; Tcd, complete decomposition time.

## Data Availability

The data presented in this study are available upon request.

## Supplementary Materials

The following are available online at https://www.mdpi.com/1999-4923/13/2/133/s1, Table S1: Properties of four HA dermal fillers (except for 99 fill^®^*); Text S1: The NONMEM 7.4 code of the kinetic model of Neuramis^®^ VOLUME LIDOCAINE; Figure S1: The goodness of fit plots of the final filler’s models (A) 99 fill^®^; (B) Juvederm^®^ VOLUMA with Lidocaine; (C) Neuramis^®^ VOLUME Lidocaine; (D) Restylane^®^ Lyft with Lidocaine; and (E) YVOIRE^®^ Contour plus.
