# Supplementary material for: Model-Based Prediction to Evaluate Residence Time of Hyaluronic Acid Based Dermal Fillers
Source: Pharmaceutics. 2021 Jan 21;13(2):133. doi: 10.3390/pharmaceutics13020133 (PMC7909806; doi:10.3390/pharmaceutics13020133)
Supplement: Supplementary file 1 [file pharmaceutics-13-00133-s001.pdf]

pharmaceutics

MDPI

Supplementary Materials: Model-Based Prediction to Evaluate Residence Time of Hyalu-ronic Acid Based Dermal Fillers

Hyo-jeong Ryu, Seong-sung Kwak, Chang-hoon Rhee, Gi-hyeok Yang, Hwi-yeol Yun and Won-ho Kang

Table S1. Properties of four HA dermal fillers (except for 99 fill®*).

Variable	Juvederm® VOLUMA with Lidocaine [1]	Neuramis® VOLUME Lidocaine [2]	Restylane® Lyft with Lidocaine [2]	YVOIRE® Contour plus [3,4]
Manufacturer	Allergan Plc.	Medytox Inc.	Galderma S.A.	LG Chem Ltd.
HA molecular weight (Da)	2.5×10^6	1.0×10^6	1.0×10^6	3.0×10^6
Cross-linker	BDDE	BDDE	BDDE	BDDE
Gel phase	Mono-phasic	Mono-phasic	Bi-phasic	Bi-phasic
HA content (mg/mL)	22–26	20	20	20

*The properties of 99 fill® could not be found due to confidentiality issues.

HA, hyaluronic acid; BDDE, 1,4-butanediol diglycidyl ether.

Text S1. The NONMEM 7.4 code of the kinetic model of Neuramis® VOLUME LIDOCAINE

```
$PROB NVL_1comp
$DATA NVL_dataset.csv IGNORE=@
$INPUT ID TIME AMT DV MDV

$SUBROUTINE ADVAN6 TOL=8

$MODEL
COMP(DEPOT, DEFDOSE)
COMP(SC, DEFOBS)

$PK
KSWELL = THETA(1) * EXP(ETA(1))
KDEG      = THETA(2) * EXP(THETA(3)*ETA(1))

$DES
DADT(1)=-KSWELL*A(1)
DADT(2)=KSWELL*A(1)-KDEG*A(2)

$ERROR
  IPRED = A(2)
  DEL = 0
  IF(IPRED.EQ.0) DEL=1
  W = IPRED+DEL
  IRES    = DV - IPRED
  IWRES = IRES/W
  Y          = IPRED + W * EPS(1)

$THETA
  (0, 0.0036)                ;KSWELL
  (0, 2.2)                   ;KDEG
  (0, 1.0056)                  ;SLOPE


$OMEGA
  0.05            ; ETA1 on KSWELL


$SIGMA
  0.1

$ESTIMATION NOABORT MAXEVAL=9999 METHOD=1 INTER PRINT=10 NSIG=2 SIGL=8
```

$TABLE ID TIME AMT DV MDV IPRED CWRES IWRES ONEHEADER NOPRINT FILE = sdtab1

$TABLE ID ETA1 ONEHEADER NOPRINT NOAPPEND FILE = patab1

$COV UNCONDITIONAL SIGL=12 PRINT=E

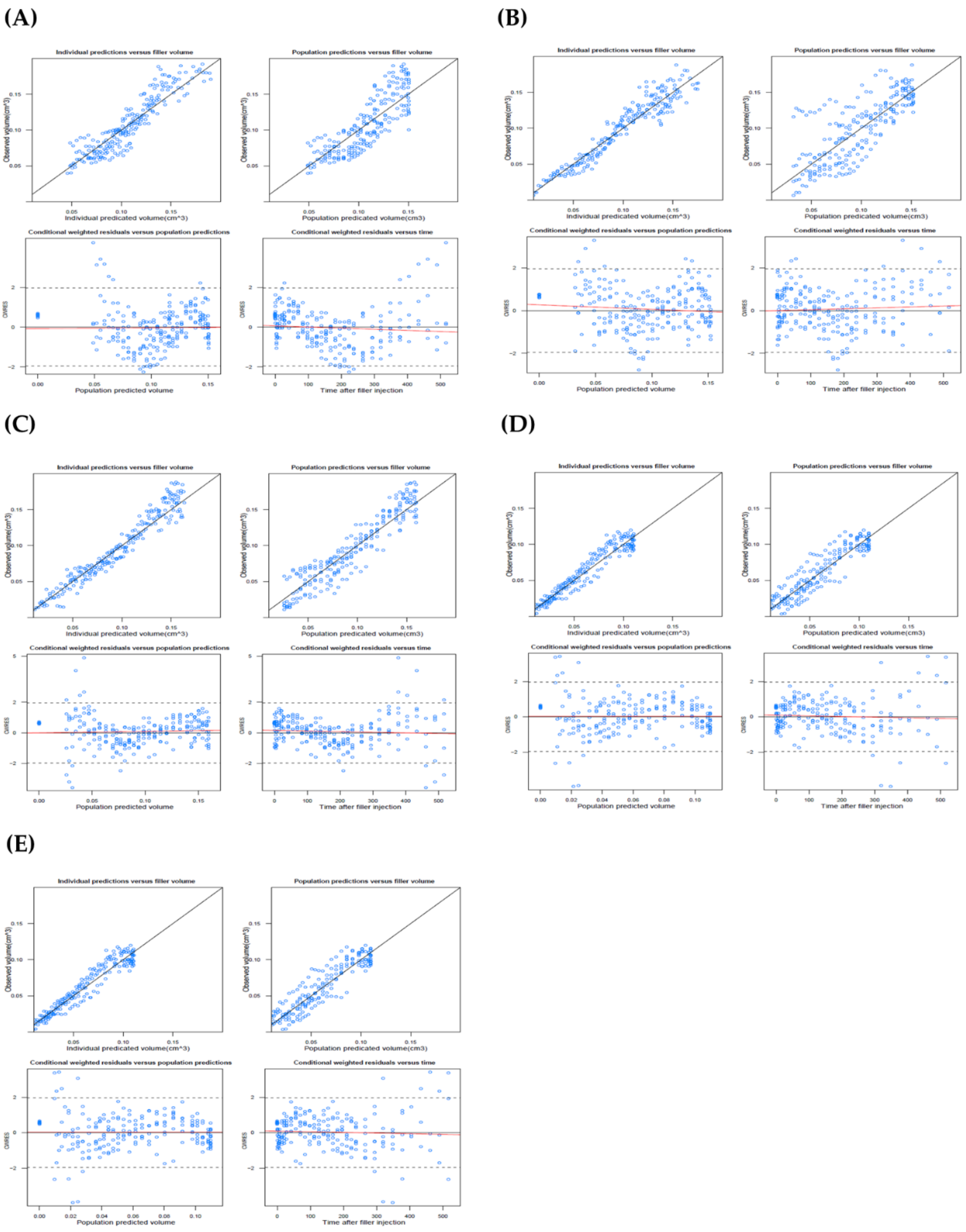

Figure S1. The goodness of fit plots of the final filler's models (**A**) 99 fill®; (**B**) Juvederm® VOLUMA with Lidocaine; (**C**) Neuramis® VOLUME Lidocaine; (**D**) Restylane® Lyft with Lidocaine; and (**E**) YVOIRE® Contour plus.

References

1. accessdata.fda.gov. Available online: https://www.accessdata.fda.gov/cdrh_docs/pdf5/P050047b.pdf (accessed on 11 January 2021).
2. Pak, C.; Park, J.; Hong, J.; Jeong, J.; Bang, S.; Heo, C.Y. A Phase III, Randomized, Multi-Center, Double-Masked, Matched-Pairs, Active-Controlled Trial to Compare the Efficacy and Safety between Neuramis Deep and Restylane in the Correction of Nasolabial Folds. *Arch. Plast. Surg.* 2015, *42*, 721–728, doi:10.5999/aps.2015.42.6.721.
3. Yang, S.D.; Shin, S.; Lee, J. Two Randomized Controlled Trials of Hyaluronic Acid Fillers for the Correction of Nasolabial Folds. *Plast. Reconstr. Surg. Glob. Open* 2020, *8*, e2975, doi:10.1097/GOX.0000000000002975.
4. YVOIRE|Medical Device|LG Chem Life Sciences Company. Available online: https://www.lgchem.com/product/PD00000220 (accessed on 11 January 2021).
